# Independent replication of polymorphisms predicting toxicity in breast cancer patients randomized between dose-dense and docetaxel-containing adjuvant chemotherapy

**DOI:** 10.18632/oncotarget.22697

**Published:** 2017-11-27

**Authors:** Annelot G.J. van Rossum, Marleen Kok, Danielle McCool, Mark Opdam, Nienke C. Miltenburg, Ingrid A.M. Mandjes, Elise van Leeuwen-Stok, Alex L.T. Imholz, Johanneke E.A. Portielje, Monique M.E.M. Bos, Aart van Bochove, Erik van Werkhoven, Marjanka K. Schmidt, Hendrika M. Oosterkamp, Sabine C. Linn

**Affiliations:** ^1^ Division of Molecular Pathology, Netherlands Cancer Institute, Amsterdam, The Netherlands; ^2^ Division of Immunology, Netherlands Cancer Institute, Amsterdam, The Netherlands; ^3^ Department of Medical Oncology, Netherlands Cancer Institute, Amsterdam, The Netherlands; ^4^ Department of Neurology, Medical Center Slotervaart, Amsterdam, The Netherlands; ^5^ Data Center, Netherlands Cancer Institute, Amsterdam, The Netherlands; ^6^ Dutch Breast Cancer Research Group, BOOG Study Center, Amsterdam, The Netherlands; ^7^ Department of Medical Oncology, Deventer Ziekenhuis, Deventer, The Netherlands; ^8^ Department of Medical Oncology, HagaZiekenhuis, The Hague, The Netherlands; ^9^ Department of Medical Oncology, Reinier de Graaf Groep, Delft, The Netherlands; ^10^ Department of Medical Oncology, Zaans Medisch Centrum, Zaandam, The Netherlands; ^11^ Biometrics Division, Netherlands Cancer Institute, Amsterdam, The Netherlands; ^12^ Department of Medical Oncology, Haaglanden Medisch Centrum, The Hague, The Netherlands; ^13^ Department of Pathology, University Medical Center, Utrecht, The Netherlands

**Keywords:** replication, single nucleotide polymorphisms, association, toxicity, chemotherapy

## Abstract

**Introduction:**

Although pharmacogenomics has evolved substantially, a predictive test for chemotherapy toxicity is still lacking. We compared the toxicity of adjuvant dose-dense doxorubicin-cyclophosphamide (ddAC) and docetaxel-doxorubicin-cyclophosphamide (TAC) in a randomized multicenter phase III trial and replicated previously reported associations between genotypes and toxicity.

**Results:**

646 patients (97%) were evaluable for toxicity (grade 2 and higher). Whereas AN was more frequent after ddAC (*P* < 0.001), TAC treated patients more often had PNP (*P* < 0.001). We could replicate 2 previously reported associations: TECTA (rs1829; OR 4.18, 95% CI 1.84-9.51, *P =* 0.001) with PNP, and GSTP1 (rs1138272; OR 2.04, 95% CI 1.13-3.68, *P =* 0.018) with PNP.

**Materials and methods:**

Patients with pT1-3, pN0-3 breast cancer were randomized between six cycles A60C600 every 2 weeks or T75A50C500 every 3 weeks. Associations of 13 previously reported single nucleotide polymorphisms (SNPs) with the most frequent toxicities: anemia (AN), febrile neutropenia (FN) and peripheral neuropathy (PNP) were analyzed using logistic regression models.

**Conclusions:**

In this independent replication, we could replicate an association between 2 out of 13 SNPs and chemotherapy toxicities. These results warrant further validation in order to enable tailored treatment for breast cancer patients.

## INTRODUCTION

Adjuvant chemotherapy for early breast cancer has improved substantially over the past decades. [1] The introduction of two classes of drugs has been particularly important: anthracyclines and taxanes. However, treatment with these very effective drugs causes significant toxicities [2].

Anthracyclines are associated with an increased risk of nausea, vomiting, bone marrow suppression, myelodysplastic syndrome, leukemia and congestive heart failure [3, 4]. Taxanes on the other hand are associated with peripheral neuropathy, febrile neutropenia and diarrhea [5]. These toxicities may put patients at risk of unfavorable outcome [2], decrease health-related quality of life and raise health-care costs due to hospital admissions. Hence, there is a great clinical need for tests that can predict which patients will encounter significant toxicity [6].

The ultimate goal is to develop a clinical test with a short lead-time that predicts treatment-specific toxicity with high accuracy. Patients with a test result indicating substantial toxicity may be spared from these side effects when an alternative systemic treatment would be prescribed. To date, numerous associations between toxicity of anthracyclines and taxanes and single nucleotide polymorphisms (SNPs) have been described [7–29]. These SNPs usually reside in genes that encode for the enzymes involved in the pharmacokinetics of these drugs. Despite plausible biological rationales, none of these associations were validated in independent studies and incorporated into clinical practice. Proper validation could have been hampered due to the methodological limitations of these studies [30]. Studies were often retrospective series instead of randomized trials with relatively small sample sizes. Moreover, these studies evaluated multiple associations, thereby increasing the risk of type I errors (false positive findings).

Here we present the toxicity of a multicenter randomized phase III trial of six cycles of dose-dense doxorubicin/cyclophosphamide (ddAC) and docetaxel/doxorubicin/cyclophosphamide (TAC). Additionally, we aim to replicate previously reported associations between side effects and clinical variables or SNPs. To our knowledge, this is the first trial that investigates 6 cycles of ddAC instead of 4. Moreover, it is the first replication of reported associations between genotype and chemotherapy toxicity in a large independent dataset including a randomization between two adjuvant regimens for breast cancer treatment.

## RESULTS

### Clinicopathological characteristics

**Figure 1:**
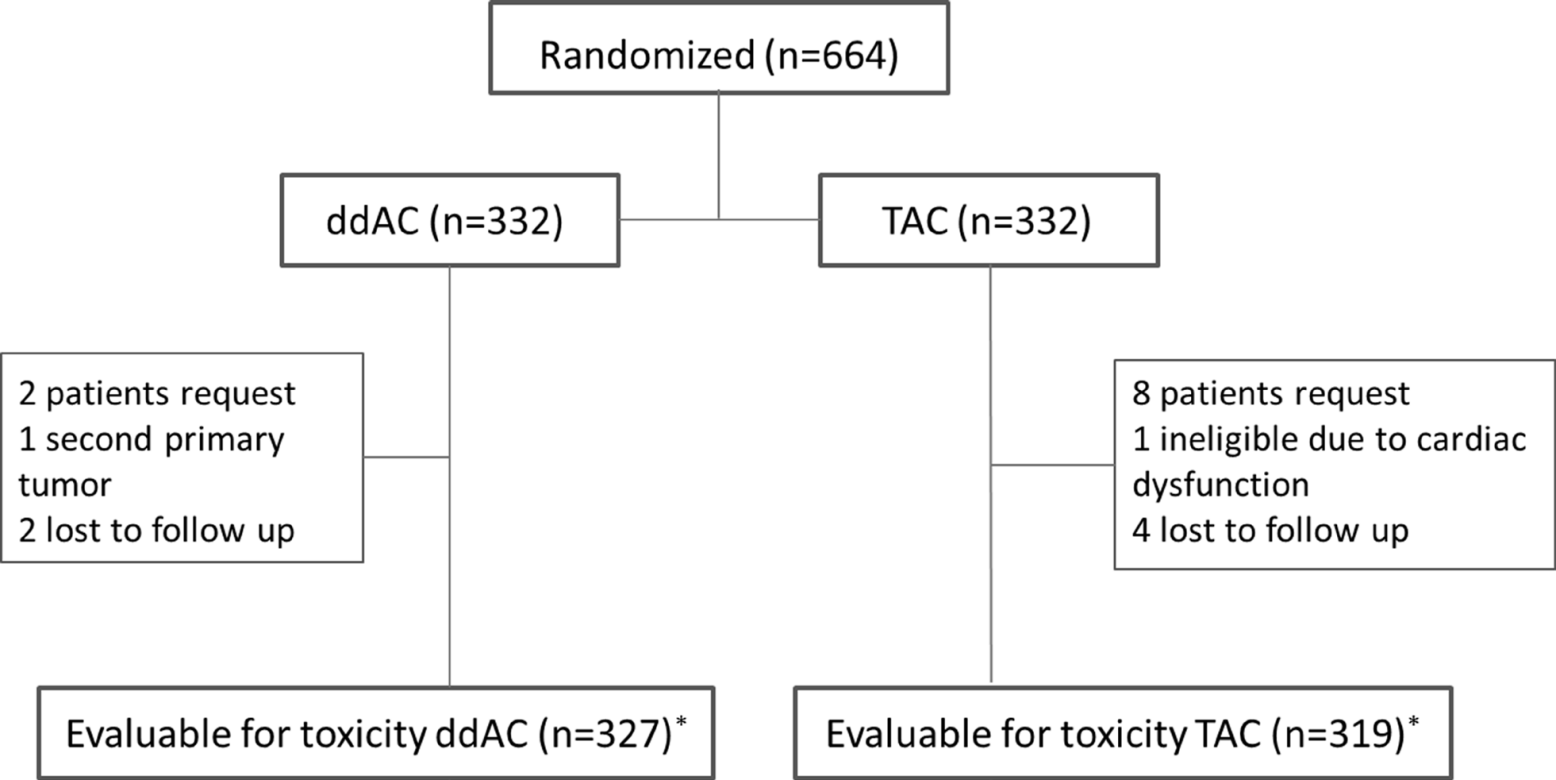
Flow chart of patients evaluable for toxicity * received at least one cycle of allocated treatment; ddAC = dose-dense doxorubicin, cyclophosphamide; TAC = docetaxel, doxorubicin, cyclophosphamide

Between August 2004 and November 2012, 664 patients were randomized (Figure 1). Sixteen patients were excluded after randomization on their own request or lost to follow up. Two patients were considered ineligible for other reasons: one patient had a second primary tumor and one patient had significant cardiac dysfunction at baseline. In total, 646 patients were evaluable for toxicity.

The two treatment groups were not significantly different according to clinicopathological characteristics (Table 1). After the introduction of trastuzumab in routine clinical practice, patients with a HER2-positive tumor were no longer eligible and consequently only a small proportion of the patients included in this trial had HER2-positive disease.

**Table 1:** Patient characteristics

	dose dense AC *n* (%)	TAC *n* (%)	Total *n* (%)	*p*-value*
Age (yrs)				0.667
≤ 29	2 (0.6)	1 (0.3)	3 (0.5)	
30-39	23 (7.0)	25 (7.8)	48 (7.4)	
40-49	115 (35.2)	123 (38.6)	238 (36.8)	
50-59	125 (38.2)	115 (36.1)	240 (37.2)	
60-69	62 (19.0)	53 (16.6)	115 (17.8)	
≥ 70	0	2 (0.6)	2 (0.3)	
Menopausal status^†^				0.323
premenopausal	168 (51.4)	175 (54.9)	343 (53.1)	
postmenopausal	154 (47.1)	137 (42.9)	291 (45.0)	
missing	5 (1.5)	7 (2.2)	12 (1.9)	
Surgery				0.490
breast conserving surgery	178 (54.4)	165 (51.7)	343 (53.1)	
mastectomy	148 (45.3)	153 (48.0)	301 (46.6)	
missing	1 (0.3)	1 (0.3)	2 (0.3)	
Endocrine therapy				0.934
none	55 (16.8)	57 (17.9)	102 (15.8)	
tamoxifen	76 (23.2)	69 (21.6)	145 (22.4)	
aromatase inhibitor	26 (8.0)	28 (8.8)	54 (8.4)	
sequential tamoxifen-aromatase inhibitor	170 (52.0)	164 (51.4)	334 (51.7)	
missing	0 (0.0)	1 (0.3)	1 (0.2)	
T Stage^‡^				0.691
T1	157 (48.0)	151 (47.3)	308 (47.7)	
T2	152 (46.5)	148 (46.4)	300 (46.4)	
T3	16 (4.9)	18 (5.6)	34 (5.3)	
T4	2 (0.6)	0	2 (0.3)	
Tx	0	1 (0.3)	1 (0.2)	
missing	0	1 (0.3)	1 (0.2)	
N Stage^‡^				0.918
N0	61 (18.7)	61 (19.1)	122 (18.9)	
N1	207 (63.3)	195 (61.1)	402 (62.2)	
N2	44 (13.5)	44 (13.8)	88 (13.6)	
N3	15 (4.6)	18 (5.6)	33 (5.1)	
missing	0	1 (0.3)	1 (0.2)	
Histology				0.310
ductal	269 (82.3)	254 (79.6)	523 (81.0)	
lobular	46 (14.1)	45 (14.1)	91 (14.1)	
other	12 (3.7)	20 (6.3)	32 (5.0)	
Grade^§^				0.480
good	32 (9.8)	40 (12.5)	72 (11.1)	
intermediate	155 (47.4)	141 (44.2)	296 (45.8)	
poor	140 (42.8)	138 (43.3)	278 (43.0)	
Subtype^¶^				0.666
ER and/or PR positive, HER2 negative	267 (81.6)	258 (80.9)	525 (81.3)	
HER2 positive	12 (3.7)	11 (3.4)	23 (3.5)	
Triple negative	48 (14.7)	50 (15.7)	98 (15.2)	

A = doxorubicin; C = cyclophosphamide; T=docetaxel § Subtypes were defined as 1. estrogen receptor (ER) and/or progesterone receptor (PR) positive, human epidermal growth factor receptor 2 (HER2) negative; 2. HER2 positive, regardless of ER or PR status; 3. Triple (ER, PR, HER2) negative; ^*^ Pearson Chi-square test or Fisher’s exact test (2-sided), missing values excluded; ^†^ Menopausal status was based on patients’ history; ^‡^ According to AJCC staging 6^th^ edition ^¶^ Grading according to the modified Bloom-Richardson grading system^33^.

### Dose reductions and delays

A total of 280 out of 327 patients randomized to ddAC (85.6%) and 271 out of 319 patients randomized to TAC (85.0%) received 6 full-dosed cycles of treatment (*P* = 0.809). For the patients who prematurely stopped treatment, ddAC was discontinued due to toxicity in 22 out of 327 patients (6.7%) and TAC in 26 out of 319 patients (8.2%, *P* = 0.491; Supplementary Table 1). Dose reductions of more than 10% occurred more frequently for TAC (39 out of 1817 cycles, 2.1%) than for ddAC (13 out of 1914 cycles, 0.7%, *P* < 0.001).

### Adverse events (AEs)

Supplementary Table 2 shows all AEs (grade 2 or higher) per treatment arm per CTCAE category.

Table 2 shows the toxicities that were significantly different between the treatment groups. Anemia was observed more often in the ddAC group than in the TAC group: 62 out of 327 patients (19.0%) versus 15 out of 319 patients (4.7%) respectively (*P* < 0.001). Also, hand-foot syndrome (4.3% vs 0.6%, *P* = 0.004), cough (5.8% vs 2.2%, *P* = 0.019) and phlebitis (4.3% vs 1.3%, *P* = 0.029) were observed more often in the ddAC treated patients.

**Table 2:** Toxicities (grade 2 or higher) with significantly different frequencies in the treatment groups

	Total *n =* 646 (%)	dose dense AC *n =* 327 (%)	TAC *n =* 319 (%)	*p*-value^*^
Anemia	77 (11.9)	62 (19.0)	15 (4.7)	< 0.001
Hand-foot syndrome	16 (2.5)	14 (4.3)	2 (0.6)	0.004^†^
Diarrhea	74 (11.5)	21 (6.4)	53 (16.6)	< 0.001
Edema limb	16 (2.5)	1 (0.3)	15 (4.7)	< 0.001^†^
Peripheral neuropathy	61 (9.4)	15 (4.6)	46 (14.4)	< 0.001
Cough	26 (4.0)	19 (5.8)	7 (2.2)	0.019
Phlebitis	18 (2.8)	14 (4.3)	4 (1.3)	0.029^†^

^*^ Pearson Chi-square test 2-sided; ^†^ Fisher’s exact test 2-sided; A = doxorubicin; C = cyclophosphamide; T = docetaxel.

Peripheral neuropathy was seen in 46 out of 319 patients (14.4%) in the TAC treatment group and in 15 out of 327 ddAC treated patients (4.6%; *P* < 0.001). In addition, diarrhea was observed more often in patients treated with TAC (16.6%) than in patients treated with ddAC (6.4%; *P* < 0.001), as was edema of the limbs (4.7% vs 0.3%; *P* < 0.001).

Of note, febrile neutropenia was observed in 36 out of 327 patients treated with ddAC (11.0%) and 40 out of 319 patients treated with TAC (12.5%) which was not significantly different (*P* = 0.546).

### Serious adverse events (SAEs)

Two patients were diagnosed with acute myeloid leukemia during follow up, one in the ddAC group and one in the TAC group (Supplementary Table 3). One ddAC treated patient developed myelodysplasia. Two TAC treated patients and one ddAC treated patient, all without known cardiovascular history, developed grade 3 or 4 symptoms of heart failure.

In total, 130 out of 646 patients (20.1%) experienced at least one SAE: 60 out of 327 patients (18.3%) in the ddAC treated group and 70 out of 319 patients (21.9%) in the TAC treated group (*P* = 0.255). Admission to the hospital due to a SAE was needed at least once in 121 patients: 55 of 327 ddAC treated patients (16.8%) and 66 of 319 TAC treated patients (20.7%; *P* = 0.207). Although there was no difference in the frequency of febrile neutropenia between the ddAC group and the TAC group, the first episode was on average after 3.7 cycles of ddAC and 1.4 cycles of TAC (*P* < 0.001).

### Single nucleotide polymorphisms (SNPs)

#### Replication of associations between clinicopathologic variables, SNPs and toxicity

**Figure 2:**
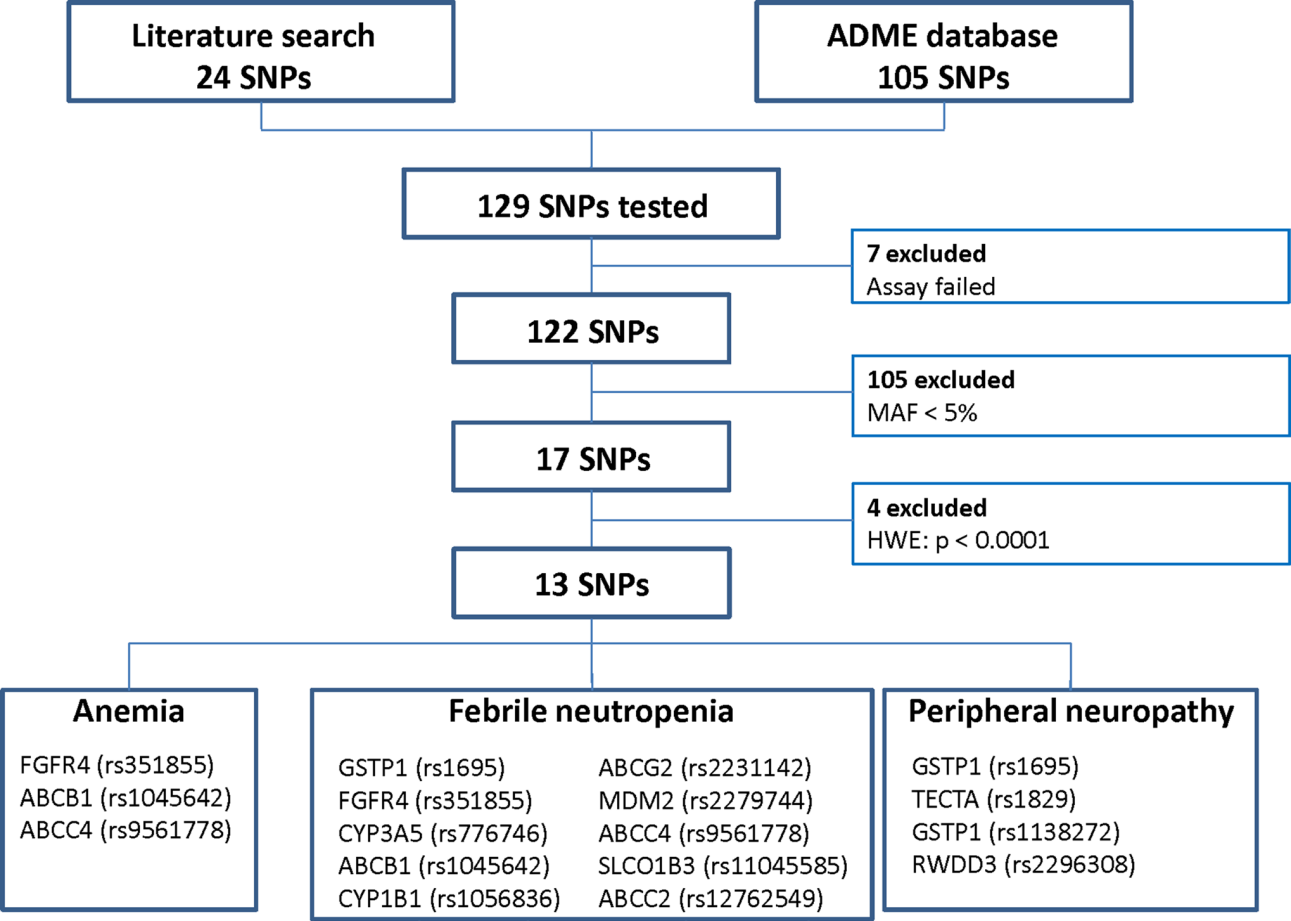
Flow chart of single nucleotide polymorphisms (SNPs) that were selected for association analyses MAF = minor allele frequency, HWE = Hardy-Weinberg Equilibrium.

We aimed to replicate previously reported associations between clinicopathologic variables or SNPs and toxicity. SNPs were selected if they were associated previously with toxicity of one of the treatment agents or if they were involved in the metabolism of one of the treatment agents (Figure 2). The results are listed in Supplementary Table 5, the significant findings are listed in Table 3.

**Table 3:** Significant associations between toxicities and clinical variables or SNPs

Toxicity	Variable	Groups	All patients			ddAC	TAC	TAC vs ddAC		Test for interaction
			No. of patients with toxicity (%)	OR (95% CI)	p-value	No. of patients with toxicity (%)	No. of patients with toxicity (%)	OR (95% CI)	*p*-value	*p*-value
**AN**	Age^9^	< 65 years vs ≥ 65 years	68/616 (11.0) 9/30 (30.0)	**3.45 (1.52-7.85)**	**0.003**	56/310 (18.1) 6/17 (35.3)	12/306 (3.9) 3/13 (23.1)	0.19 (0.10-0.35) **0.55 (0.11-2.81)**	**< 0.001** 0.472	0.223
**AN**	Baseline platelet count9	> 200x109 cells/L vs ≤ 200x109 cells/L	63/585 (10.8) 14/55 (25.5)	**2.83 (1.46-5.48)**	**0.002**	52/294 (17.7) 10/28 (35.7)	11/291 (3.8) 4/27 (14.8)	0.18 (0.09-0.36) 0.31 (0.08-1.16)	< 0.001 0.083	0.475
**FN**	FGFR4 (rs351855)	CC/CT vs TT	66/579 (11.4) 8/59 (13.6)	1.22 (0.55-2.68)	0.622	28/293 (9.6) 7/30 (23.3)	38/286 (13.3) 1/29 (3.4)	1.45 (0.86-2.43) 0.12 (0.01-1.02)	0.160 0.053	0.027
**PNP**	TECTA(rs1829)28	CC/CT vs TT	52/608 (8.6) 9/32 (28.1)	**4.18 (1.84-9.51)**	**0.001**	14/315 (4.4) 1/9 (11.1)	38/293 (13.0) 8/23 (34.8)	**3.20 (1.70-6.05)** **4.27 (0.45-40.44)**	< 0.001 0.206	0.810
**PNP**	GSTP1 (rs1138272)31	CC vs CT/TT	43/525 (8.2) 18/117 (15.4)	**2.04 (1.13-3.68)**	**0.018**	11/275 (4.0) 4/50 (8.0)	32/250 (12.8) 14/67 (20.9)	**3.52 (1.74-7.15)** **3.04 (0.93-9.88)**	< 0.001 0.065	0.833

ddAC = dose-dense doxorubicin, cyclophosphamide; TAC = docetaxel, doxorubicin, cyclophosphamide; OR = odds ratio; CI = confidence interval.

### Anemia (AN) [7–10]

The odds of anemia in patients who were 65 years or older was 3.45 times the odds in the younger patients (30% vs 11%, *P* = 0.003) (Table 3). Baseline platelet count of 200 × 10^9^ cells/L or less was also associated with higher risk of anemia (25.5% vs 10.8%, *P* = 0.002). Previously reported genotypes for *FGFR4* (CC vs CT/TT) [8], *ABCB1* (TT/TC vs CC) [9] and *ABCC4* (GG vs GT/TT) [10] were not significantly associated with anemia in our dataset (Supplementary Table 6). The associations of age and baseline platelet count with anemia remained stable in a multivariable model.

### Febrile neutropenia (FN) [9, 11–25]

Baseline absolute neutrophil count (ANC ≤ 3.1 × 10^9^ cells/L)(15) and the following previously reported genotypes did not have a significant association with FN: *GSTP1* (AG rs1695 and CC rs1138272 vs other; rs1695 AA vs AG/GG)(18,19), *ABCB1* (TT vs TC/CC)(9,17), *ABCG2* (CC vs CA/AA)(22), *MDM2* (TT/TG vs GG)(23), *ABCC4* (GG vs GT/TT)(24), *SLCO1B3* (AA vs AG/GG)(25) and *ABCC2* (CC/CG vs GG)(25) and a haplotype of *ABCB1* and *CYP1B1* (rs1045642*rs1056836)(21) (Supplementary Table 6).

### Peripheral neuropathy (PNP) [26–29]

The odds of PNP in homozygous variant carriers of *TECTA* (TT, rs1829) was 4.18 times increased compared with the odds in homozygous wildtype or heterozygous variant carriers (CC/CT) in our cohort (28.1% vs 8.6%, *P* = 0.001) (Table 3). In addition, heterozygous and homozygous variant carriers of *GSTP1* (CT/TT, rs1138272) had 2.04 times increased odds of PNP (15.4% vs 8.2%, *P* = 0.018). In our dataset, a history of diabetes as previously described by Bhatnagar et al [27], was not related with PNP (Supplementary Table 6). Also, previously reported genotype subgroups for *GSTP1* (AA vs AG/GG) [28] and *RWDD3* (GG/GT vs TT) [26] were not significantly associated with PNP.

### SNPs and differential toxicity of ddAC or TAC

Next, we evaluated whether the associations between the SNPs and toxicities of interest were different in the two treatment arms. The significant tests for interaction of the treatment effect are included in Table 3.

### Anemia

We found no significant interaction between a clinical variable or SNP and treatment (ddAC vs TAC) for the risk of developing anemia (Supplementary Table 7).

### Febrile neutropenia

Although treatment was not significantly associated with toxicity in the *FGFR4* (rs351855) genotype subgroups, we did observe a significant interaction between treatment and this SNP (*P* = 0.027, Table 3 and Supplementary Figure 2). Interaction analyses of other clinical variables or SNPs with FN were not significant. Of note, AG carriers of rs1695 and CC carriers of rs1138272 in *GSTP1* had a significantly higher risk of FN when treated with TAC (OR 2.14, 95% CI 1.08–4.23, *P* = 0.029), which was not observed in the ddAC treated group (OR 0.98, 95% CI 0.47–2.05, *P* = 0.959).

### Peripheral neuropathy

None of the investigated factors had a significant interaction with treatment on the risk of PNP.

## DISCUSSION

The main objective of the research presented here was to replicate previously described associations between certain clinical parameters or genetic polymorphisms and three frequently observed and clinically important chemotherapy-induced toxicities. Regarding the clinical parameters, we were able to replicate the associations of age and baseline platelet count with risk of anemia as previously described by Dranitsaris et al [7]. Of the 13 SNPs tested, the variant genotypes of rs1829 in *TECTA* and rs1138272 in *GSTP1* were related to peripheral neuropathy. However, the test for interaction between use of docetaxel, these variant genotypes and PNP was not significant. Given the relatively low sample size of our study, validation is required to determine the clinical value of our findings.

Most previously described associations could not be replicated in our study. This might be due to the fact that these associations were often described in patients treated with a different regimen than the agents used in our study. Also, previously described associations could have been incidental findings in inadequately designed studies. Instead of taking an agnostic approach in evaluating the predictive value of numerous SNPs, we focused on already described associations between genotype and frequently occurring side effects. With this starting point we reduce the type I error (false positive findings). The randomized nature of our dataset allowed us to evaluate whether these associations are treatment-specific and could therefore be of use in tailoring adjuvant chemotherapy for breast cancer patients. None of the SNPs in the different toxicity models were in linkage disequilibrium, except for a minor linkage between both *GSTP1* SNPs (rs1695 and rs1138272; r^2^ 0.162), indicating that we investigated independent SNPs.

The largest difference in risk of toxicity was observed for *TECTA*. Homozygous variant carriers of *TECTA* (rs1829) had an increased risk of PNP. The mechanistic explanation regarding the link between *TECTA* and PNP is elusive. Tectorin Alpha (TECTA) is a major component of the tectorial membrane in the inner ear, which is important for transducing sound into electrical signals for our nervous system. Mutations in the *TECTA* gene are therefore often linked to deafness [31]. Our findings are in line with the preliminary findings of Schneider et al [26], who reported an association between *TECTA* polymorphism and taxane-induced-PNP. However, in the final report of Schneider et al [32] and two other genome wide association studies [33, 34] the association could not be replicated. In addition, in our study the association between treatment and PNP was not significantly different in the *TECTA* genotype subgroups as tested by the interaction analysis. This might be due to the relatively small sample size and an imbalance in the distribution of *TECTA* genotypes between the treatment arms. Alternatively, *TECTA* homozygous variant alleles may be associated with higher vulnerability of nerve tissues to cytotoxic damage in general. In the latter case, *TECTA* genotype analysis might only appear valuable when balancing risks and benefits of adjuvant chemotherapy in an equivocal case where PNP might be detrimental (e.g. a professional violin player). However, before introducing *TECTA* genotype analysis in daily clinical practice, these data require validation in an independent, large, preferably prospective cohort using the exact same subgrouping of patients according to their genotype.

To explore potential tailored chemotherapy based on SNP analysis, we tested the effect of treatment on the risk of toxicity in the genotype-based patient subgroups by performing an interaction analysis. The risk of FN according to *FGFR4* genotype was significantly different in the ddAC subgroup compared to the TAC subgroup as determined by the test for interaction. However, the absolute number of patients in the investigated subgroups is very small and an explanation for the opposite effect in the TAC arm versus the ddAC arm is lacking. Moreover, the mechanism by which a polymorphism of fibroblast growth factor receptor 4 (*FGFR4*) can lead to an increased risk of FN is unknown. Therefore, the observed interaction between this *FGFR4* variant, treatment and FN should be considered hypothesis-generating.

To our knowledge, this is the first report describing toxicity data of 6 cycles of adjuvant ddAC in high risk breast cancer patients in the context of a multicenter phase III randomized trial. In the ddAC treated subgroup as well as the TAC treated subgroup, 85% of the patients received 6 full-dosed cycles of treatment. Compared with 4 cycles of ddAC as described by Jones et al [35], anemia was more frequently observed in our ddAC treated cohort (19% vs 7%, resp.) suggesting that this might be related to the two additional cycles of ddAC. Indeed, 32 out of 62 occurrences of anemia (52%) were observed in cycles 5 and 6. In addition, the prevalence of anemia after 6 cycles of AC in the Cancer and Leukemia Group B 40101 trial (6%) was also less than in our cohort [36], indicating that the combination of the dose dense schedule and two additional cycles cause an increased frequency of anemia. In line with the observations by Jones [35], we observed febrile neutropenia in 11% of the patients during six cycles of ddAC, despite the use of G-CSF. In the CALGB 40101 cohort [36], febrile neutropenia was seen in only 6% of the patients. Although these comparisons are indirect, it suggests that the dose dense schedule has a considerable effect on the incidence of FN. As observed rarely in the CALGB 40101 trial (AC, <1%) [36] and during a single institution trial evaluating FAC (10%) [37] , also ddAC treated patients encountered PNP, which might be related to cyclophosphamide. For our TAC treated subgroup, we compared our results with adverse events in patients receiving equally dosed TAC in the GeparTrio trial and the Breast Cancer International Research Group (BCIRG) trial 001 [38, 39]. Whereas 1.3% of the GeparTrio trial patients had grade 3–4 neuropathy and up to 47.1% had any grade of neuropathy, PNP grade 2 or higher was observed in 14.4% of our TAC treated patients. In the BCIRG 001, 3.6% of the patients treated with TAC had neurosensory effects grade 2 or higher and 25.5% had neurosensory effects of any grade. The incidence of heart failure (ddAC 0.3%, TAC 0.6%) and leukemia or myelodysplastic syndrome (ddAC 0.6%, TAC 0.6%) was low in our study.

This study has some limitations. Most GWAS and SNP association studies use germline DNA from normal tissue, often peripheral blood cells. In our cohort, normal tissue was available in only 25% of the patients, the remainder 75% was based on FFPE tumor tissue. In line with a previous report on genotype classifications in tumor tissue and normal tissue [40], concordance of 19 SNP genotypes, including the 13 selected SNPs, on 15 pairs of tumor tissue and normal tissue of our cohort was 93-100%. Likewise, concordance on 20 pairs of fresh frozen tumor tissue and FFPE tumor tissue was 94–100%. Although similarity is high, we cannot exclude that we had some misclassification of genotypes, especially for those assays that were excluded due to violation of the Hardy-Weinberg equilibrium and whose genotype distribution deviated from what was reported in the Database of Single Nucleotide Polymorphisms (dbSNP; http://www.ncbi.nlm.nih.gov/snp/). However, importantly, MAFs of the 13 SNPs were in line with those reported in dbSNP. In addition, all 13 SNPs were in Hardy-Weinberg equilibrium and as expected there was no correlation between type of tissue (normal vs tumor) used for analyses and AN, FN and PNP respectively (data not shown). These observations support the idea that the type of tissue does not seem to have a significant influence on the genotype calls of the 13 SNPs included in our analyses.

Secondly, frequencies of genetic variants, including ADME genes, are related to ethnic origin [32, 41]. Therefore, many association studies take ethnicity into account. Unfortunately, we did not have data on ethnic origin. However, the study was conducted across the Netherlands, in a probably mainly Caucasian population. Moreover, since the European population has relatively low diversity in functionally important ADME genes [41], it is unlikely that ethnic background has influenced these findings to a relevant extent.

Thirdly, the sample size of our cohort is limited. The original randomized trial was powered to define a gene expression profile predictive of recurrence free survival benefit of either of the two treatments. Because of limited power, we selected only three commonly observed toxicities to test for associations with SNPs. However, when split by treatment and subsequently by genotype subgroup, the numbers of patients who encountered any of these toxicities are low. Our data should therefore be assessed as contributing to existing evidence and hypothesis-generating.

Finally, methods used in this study may deviate from the methods of the previously reported association studies. Treatments might differ with regard to the combination of agents, the number of cycles and the schedule of administration. Besides, grades of the reported toxicity or endpoints might vary between studies. These distinct methods hamper replication of the associations for some SNPs. However, an association between a SNP and toxicity that is of potential clinical relevance should be found in a variety of studies regardless of applied methods.

The strength of our study is that we analyzed a prospective randomized dataset. However, our SNP analyses were exploratory and not prespecified in primary or secondary objectives. Since our patients were not stratified for the investigated genotypes, the distribution of these variables over the treatment arms was occasionally imbalanced (e.g. genotypes of *TECTA*). However, by replicating previously reported associations instead of identifying new ones, this study contributes to expanding evidence on these associations and provides information on what the potential role is of these SNPs in clinical practice.

This randomized study allowed us to directly compare the toxicity profile of 6 cycles of ddAC and TAC and replicate previously reported associations between toxicities and specific genotypes. The majority of these associations were not found in our cohort. This is in line with a study on radiation toxicity and SNPs in which none of the previously reported relations could be detected in a large independent dataset [42]. However, we were able to replicate some of the associations despite the relatively limited cohort size and the unplanned nature of the analyses. Also, SNP selection was limited by the time frame of the literature search, excluding more recently published, promising associations. Validation of high priority candidate SNPs in an independent cohort or a meta-analysis is desirable and will create a solid basis for biomarker driven prospective trials. These trials are needed to facilitate the entry of robust, simple and cost-effective methods to predict chemotherapy-induced toxicities into the clinic.

## MATERIALS AND METHODS

### Study design

The MATADOR trial (Microarray Analysis in breast cancer to Tailor Adjuvant Drugs Or Regimens, ISRCTN61893718) is a prospective, multicenter, non-blinded randomized phase III trial conducted in the Netherlands during 2004-2012. Twenty-nine centers participated in this study. The primary objective of this study was to discover a gene expression profile that can predict recurrence free survival (RFS) benefit of either dose-dense or docetaxel-containing, anthracycline-based adjuvant therapy. Here we present SNP and toxicity data of this study. Female patients with a stage pT1-3, pN0-3, M0 invasive adenocarcinoma of the breast were eligible (Supplementary Figure 1). A WHO performance status of 0 or 1 and adequate bone marrow, liver and renal function were required. Patients with pre-existing motor or sensory neuropathy of grade 2 or more were ineligible, as well as patients who received previous systemic anticancer therapy. At the start of the trial, trastuzumab was not part of daily clinical practice and patients with HER2-positive disease were therefore included in this study. In February 2006 however, the protocol was amended to allow trastuzumab treatment for HER2-positive disease after completion of study treatment. In view of the accumulating evidence of improved disease free survival after concurrent chemotherapy and trastuzumab, patients with HER2-positive disease became ineligible in September 2007.

Patients were stratified according to menopausal status, type of surgery, tumor size, nodal status, hormone receptor status (estrogen receptor and progesterone receptor), HER2 status and treatment center. Subsequently, patients were allocated to receive either six cycles of doxorubicin 60 mg/m^2^ plus cyclophosphamide 600 mg/m^2^ every 2 weeks (ddAC), or six cycles of docetaxel 75 mg/m^2^, doxorubicin 50 mg/m^2^ and cyclophosphamide 500 mg/m^2^ every 3 weeks (TAC). All patients received granulocyte-colony stimulating factor (pegfilgrastim). Prophylactic antibiotic therapy was not recommended. Anti-emetic treatment was given according to the local standards. Patients received adjuvant radiotherapy and/or endocrine therapy according to the Dutch guidelines.

Toxicities were reported in the clinical record form according to common toxicity criteria for adverse events (AEs; CTCAE version 3.0). All adverse events (AE) of grade 2 or higher were recorded. Anemia was defined as a baseline hemoglobin concentration 6.2 mmol/L or less, febrile neutropenia was described as a body temperature of ≥ 38.5°C and an absolute neutrophil count of < 1.0 × 10^9^/L, and peripheral neuropathy was defined as sensory alterations, paresthesia or weakness interfering with function. Any event that was fatal, life threatening, required hospitalization, led to prolonged hospitalization or resulted in significant disability was described as a serious adverse event (SAE).

The study protocol was approved by the medical ethical committee of the Netherlands Cancer Institute (approval 24 March 2004) and the research was conducted in accordance with the Declaration of Helsinki (version 17C, 1964). All patients had given written informed consent to participate in the study, including side studies meant to improve breast cancer diagnostics or therapy.

### Tumor histology and immunohistochemistry (IHC)

Formalin-fixed paraffin-embedded (FFPE) tumor tissue was assessed for morphology, histological grade according to the modified Bloom-Richardson classification [43], expression of the estrogen receptor (ER), progesterone receptor (PR) and human epidermal growth factor receptor 2 (HER2) by the pathologists of the participating centers according to established local procedures. The Dutch guidelines specified ER and PR nucleic staining of 10% staining or more as positive. HER2 score of 3+ was considered positive. In case of a 2+ HER2 score, an *in situ* hybridization assay was performed.

Breast cancer subtype was defined as 1. ER and/or PR positive, HER2 negative; 2. HER2 positive, regardless of ER and PR status; or 3. triple negative.

### DNA isolation

Fresh frozen (FF) and FFPE tumor tissue as well as normal tissue was requested from all patients. FFPE tumor tissue was available for the majority of the cases (75%). If unavailable, FF tumor tissue (18%) or FFPE normal tissue (7%) was used. For FFPE tissue, DNA was isolated as previously described using 10 slides of 10 μm, the QIAamp DNA extraction kit and protocol (QIAgen) [44].

For FF tissue, 15 slides of 30 µm were used. DNA was isolated using the DNeasy Blood & Tissue Kit (QIAgen). DNA was available for 642 patients.

### Single nucleotide polymorphisms (SNPs)

To reduce the risk of multiple testing, three toxicity categories were selected for SNP analyses based on a combination of most frequent, largest clinical impact (hospital admission) and potentially long-term disability. These three categories were anemia (A), febrile neutropenia (FN) and peripheral neuropathy (PNP).

The SNP selection procedure is illustrated in Figure 2. First, a PubMed search was performed to select SNPs based on previously reported associations between toxicity of either doxorubicin, cyclophosphamide or docetaxel and a SNP. The literature search contained three elements: 1. one of the three toxicity categories, 2. the study drugs, and 3. single nucleotide polymorphism. SNPs associated with toxicity reported until September 2015 were selected. An update of the search was performed in June 2016. The search resulted in 24 SNPs with a possible association with toxicity. Secondly, we selected 105 SNPs that could be involved in the metabolism of one of the study drugs from the PharmaADME database (http://www.pharmaadme.org/). These two strategies resulted in a total of 129 SNPs. A SNP was excluded from further analyses if the assay failed due to technical reasons (*n* = 7), if the minor allele frequency (MAF) was below 5% (*n* = 105), or if the genotype frequencies of a SNP deviated from Hardy-Weinberg equilibrium (HWE, *P* < 0.001, *n* = 4, Supplementary Table 4). A total of 13 different SNPs were included in the final analyses. The previously reported associations between these SNPs and the toxicities were summarized in Supplementary Table 5.

A customized, mass-spectrometry based genotyping assay (Sequenom MassARRAY platform, Sequenom Inc, CA, USA) was designed to analyze these SNPs. Genotypes were determined using Sequenom’s TyperAnalyzer software.

### Statistics

Differences in clinicopathological characteristics, AEs and SAEs between treatment groups were compared with a chi-square test. When the count in any of the groups was less than 5, a Fisher’s exact test was applied.

To accurately replicate previously reported associations between clinical parameters (if a cut off was reported) or SNPs and one of the three toxicities, univariable binary logistic regression models were constructed using previously reported genotype categories. All variables that were significantly associated with toxicity in the univariable models were included in a multivariable binary logistic regression model.

Secondly, tests for interaction were performed to evaluate whether the risk of a genotype-based patient group for a particular toxicity was different per given treatment (ddAC or TAC). The association of the allocated treatment with toxicity was investigated using a logistic regression model in subgroups of patients. Interactions between clinical parameters or SNPs and treatment were tested using logistic regression model with an interaction term.

The association analyses were exploratory and were not pre-specified in the analysis plan of the MATADOR trial. Since our objective was to replicate previously described associations between SNPs and toxicity, we did not correct for multiple testing. For all analyses, a p-value of less than 0.05 was considered significant. Analyses were performed using SPSS software version 22 (IBM Corporation, Armonk, NY).

## SUPPLEMENTARY MATERIALS FIGURES AND TABLES











## Abbreviations

AN: anemia
AE: adverse event
BCIRG: Breast Cancer International Research Group
CTCAE: common toxicity criteria for adverse events
ddAC: dose dense doxorubicin/cyclophosphamide
RFS: recurrence free survival
ER: estrogen receptor
FF: fresh frozen
FFPE: formalin-fixed paraffin-embedded
FGF: fibroblast growth factor
FGFR4: fibroblast growth factor receptor 4
FN: febrile neutropenia
HER2: human epidermal growth factor receptor 2
HWE: Hardy-Weinberg equilibrium
IHC: immunohistochemistry
MAF: minor allele frequency
MATADOR: Microarray Analysis in breast cancer to Tailor Adjuvant Drugs Or Regimens
PNP: peripheral neuropathy
PR: progesterone receptor
SAE: serious adverse event
SNP: single nucleotide polymorphisms
TAC: docetaxel/doxorubicin/cyclophosphamide
TECTA: tectorin alpha
