# Supplementary material for: Independent replication of polymorphisms predicting toxicity in breast cancer patients randomized between dose-dense and docetaxel-containing adjuvant chemotherapy
Source: Oncotarget. 2017 Nov 27;8(69):113531–42. doi: 10.18632/oncotarget.22697 (PMC5768344; doi:10.18632/oncotarget.22697)
Supplement: Supplementary file 1 [file oncotarget-08-113531-s001.pdf]

Independent replication of polymorphisms predicting toxicity in breast cancer patients randomized between dose-dense and docetaxel-containing adjuvant chemotherapy

SUPPLEMENTARY MATERIALS

Supplementary Table 1: Number of treatment cycles, dose reductions of at least 10%, dose delays and discontinuation of therapy due to toxicity per treatment arm

	dose dense AC				TAC			
	**No. of cycles**	**No. of dose reductions (%)**	**Stop due to toxicity after cycle no. (%)**	**Delays due to toxicity**	**No. of cycles**	**No. of dose reductions (%)**	**Stop due to toxicity after cycle no. (%)**	**Delays due to toxicity**
**Cycle 1**	327	0	0	0	319	0	5 (1.6)	0
**Cycle 2**	326	1 (0.3)	0	9 (2.8)	310	9 (2.9)	4 (1.3)	7 (2.3)
**Cycle 3**	326	4 (1.2)	4 (1.2)	13 (4.0)	306	2 (0.7)	4 (1.3)	10 (3.3)
**Cycle 4**	321	4 (1.2)	4 (1.2)	12 (3.7)	302	8 (2.6)	5 (1.7)	6 (2.0)
**Cycle 5**	317	1 (0.3)	14 (4.4)	19 (6.0)	295	8 (2.7)	8 (2.7)	16 (5.4)
**Cycle 6**	297	3 (1.0)	0	22 (7.4)	285	12 (4.2)	0	5 (1.8)
**Total**	1914	13 (0.7)	22 (6.7)	75 (3.9)	1817	39 (2.1)	26 (8.2)	44 (2.4)

A = doxorubicin; C = cyclophosphamide; T=docetaxel

Supplementary Table 2: Number of adverse events (grade 2 or higher) for each CTCAE category

	dose dense AC<br>*n* = 327	TAC<br>*n* = 319	Total<br>*n* = 646	*p*-value*
**Allergy/Immunology**	2	7	9	0.103†
**Blood/Bone marrow**	78	41	119	< 0.001
Anemia	62 (18.9)	15 (4.7)	77 (11.9)	< 0.001
Leukocytopenia	30 (9.2)	20 (6.3)	50 (7.7)	0.167
Neutropenia	9 (2.8)	8 (2.5)	17 (2.6)	0.846
Thrombopenia	7 (2.1)	3 (0.9)	10 (1.5)	0.340†
**Cardiac Arrhythmia**	7	3	10	0.340†
**Cardiac general**	0	4 (1.3)	4 (0.6)	0.059†
**Constitutional symptoms**	130	118	248	0.470
Fatigue	117 (35.8)	109 (34.2)	226 (35.0)	0.668
Fever (without neutropenia)	14 (4.3)	10 (3.1)	24 (3.7)	0.441
**Dermatology/Skin**	118	106	224	0.446
**Endocrine**	4	9	13	0.170†
**Gastrointestinal**	125	133	258	0.368
Anorexia	19 (5.8)	9 (2.8)	28 (4.3)	0.062
Constipation	16 (5.0)	25 (7.8)	41 (6.3)	0.125
Diarrhea	21 (6.4)	53 (16.6)	74 (11.5)	< 0.001
Mucositis	15 (4.6)	11 (3.4)	26 (4.0)	0.462
Nausea	65 (20.0)	52 (16.3)	117 (18.1)	0.238
Vomiting	35 (10.7)	21 (6.6)	56 (8.7)	0.063
**Hemorrhage/Bleeding**	1	0	1	1.000†
**Hepatobilliary/Pancreas**	1	0	1	1.000†
**Infection**	94	94	188	0.840
Febrile neutropenia	36 (11.0)	40 (12.5)	76 (11.8)	0.546
**Edema limb**	1	17	18	< 0.001
**Metabolic/Laboratory**	11	8	19	0.235
**Musculoskeletal/Soft tissue**	2	2	4	1.000†
**Neurology**	32	62	94	0.001
Peripheral neuropathy	15 (4.6)	46 (14.4)	61 (9.4)	< 0.001
**Ocular/Visual**	14	11	25	0.583
**Pain**	42	49	91	0.358
Bone	8 (2.4)	13 (4.1)	21 (3.3)	0.243
Head	17 (5.2)	8 (2.5)	25 (3.9)	0.076
**Pulmonary/Upper respiratory**	48	28	76	0.020
Cough	19 (5.8)	7 (2.2)	26 (4.0)	0.019
Dyspnea	19 (5.8)	20 (6.3)	39 (6.0)	0.806
**Renal/Genitourinary**	3	0	3	0.249†
**Sexual/Reproductive system**	3	2	5	1.000†
**Syndromes**	3	4	7	0.722†
**Vascular**	21	10	31	0.051

For the CTCAE categories, the numbers reflect the number of patients that had at least one side effect in that CTCAE category. For the individual side effects (blanc rows), the observed toxicity is counted once per patient. * Pearson's chi square test (2-sided); † Fisher's exact test was applied.

Supplementary Table 3: Toxicities of special interest

	dose dense AC<br>*n* = 327	TAC<br>*n* = 319	Total<br>*n* = 646
**Acute myeloid leukemia**	1	1	2
**Myeolodysplastic syndrome**	1		1
**Heart failure grade 3-4**	1	2	3

Supplementary Table 4: Distribution of genotypes and Hardy Weinberg Equilibrium test for selected genetic variants. *Pearson chi-square test (2-sided), missing values excluded. See Supplementary_Table_4

Supplementary Table 5: Summary of the original association studies for anemia (A), febrile neutropenia (B) and peripheral neuropathy (C). See_Supplementary_Table 5

Supplementary Table 6: Validation of previously reported associations between anemia (A1), febrile neutropenia (B1) and peripheral neuropathy (C1) and SNPs using univariate binary logistic regression analyses. Multivariate binary logistic regression analyses (A2, C2) were made with only the significantly different factors. OR = odds ratio; CI = confidence interval. See Supplementary_ Table_6

Supplementary Table 7: Risk of anemia (A), febrile neutropenia (B) and peripheral neuropathy (C) per treatment arm in previously reported clinical or genotype subgroups. OR = odds ratio; CI = confidence interval. See Supplementary_Table_7

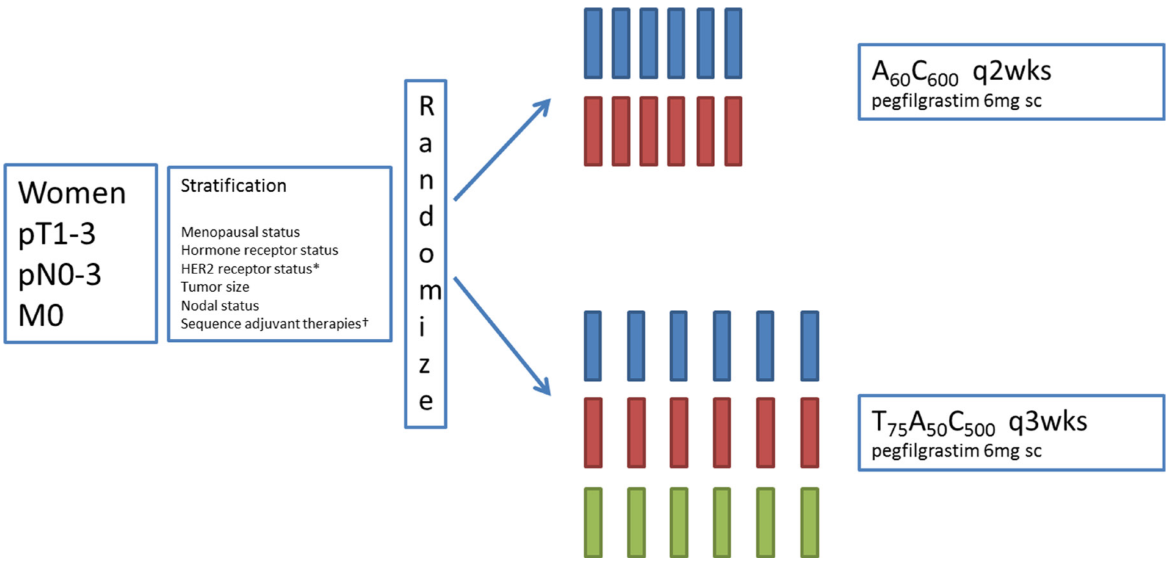

Supplementary Figure 1: Design of the Matador study: a multicenter, randomized phase III trial. *The Matador study included patients from 2004 to 2012; HER2 positive patients were included in the Matador study until August 2007, afterwards they were excluded due to perceived superiority of concurrent administration of trastuzumab with chemotherapy. †The sequence of adjuvant radiotherapy followed by chemotherapy or vice versa. HER2 = human epidermal growth factor receptor 2; A = doxorubicin; C = cyclophosphamide; T = docetaxel; wks = weeks; mg = milligram.

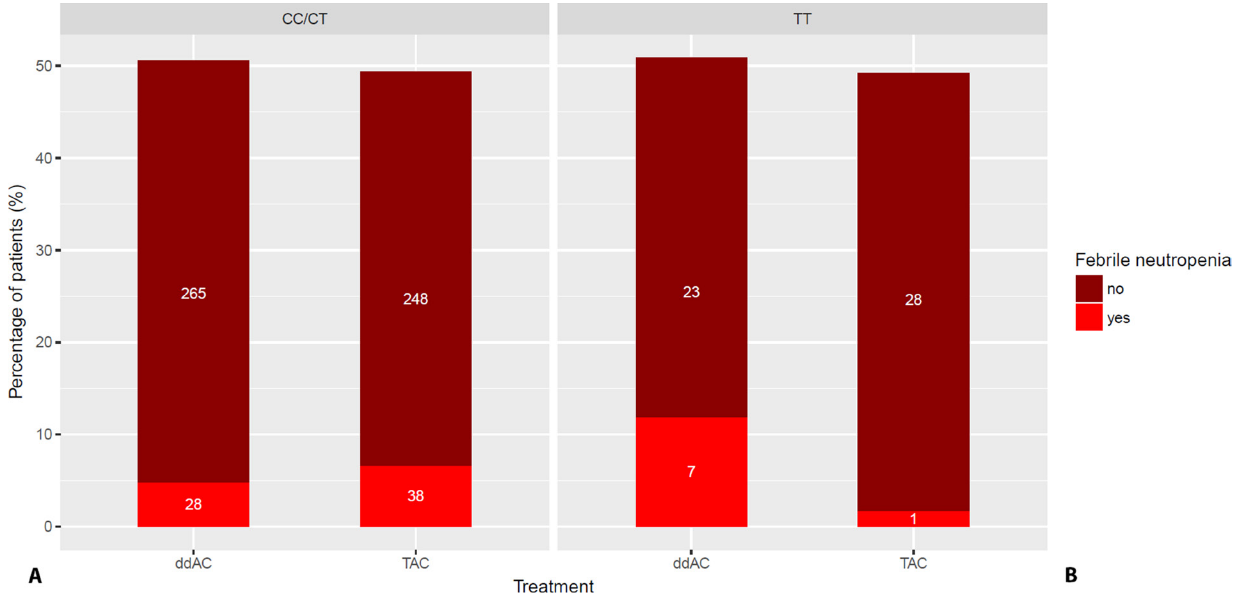

Supplementary Figure 2: Proportion of patients with febrile neutropenia per treatment arm in patients with a CC/CT genotype (**A**) or a TT genotype (**B**) for *FGFR4*. The numbers in the bars represent the number of patients.
