# Supplementary material for: Independent replication of polymorphisms predicting toxicity in breast cancer patients randomized between dose-dense and docetaxel-containing adjuvant chemotherapy
Source: Oncotarget. 2017 Nov 27;8(69):113531–42. doi: 10.18632/oncotarget.22697 (PMC5768344; doi:10.18632/oncotarget.22697)
Supplement: Supplementary file 2 [file oncotarget-08-113531-s002.docx]

**Supplementary Table 4: Distribution of genotypes and Hardy Weinberg Equilibrium test for selected genetic variants.** * Pearson chi-square test (2-sided), missing values excluded.

|  |  | Genotype | No. |  | HWE^*^ | |
| --- | --- | --- | --- | --- | --- | --- |
|  |  |  |  |  | χ2 | p-value |
| GSTP1 | rs1695 | G | 83 |  | 0.041 | 0.840 |
|  |  | AG | 297 |  |  |  |
|  |  | A | 275 |  |  |  |
|  |  | NA | 4 |  |  |  |
| TECTA | rs1829 | C | 398 |  | 0.003 | 0.960 |
|  |  | CT | 227 |  |  |  |
|  |  | T | 32 |  |  |  |
|  |  | NA | 2 |  |  |  |
| FGFR4 | rs351855 | C | 312 |  | <0.001 | 0.988 |
|  |  | CT | 280 |  |  |  |
|  |  | T | 63 |  |  |  |
|  |  | NA | 4 |  |  |  |
| CYP3A5 | rs776746 | G | 546 |  | 6.110 | 0.0134 |
|  |  | AG | 100 |  |  |  |
|  |  | A | 11 |  |  |  |
|  |  | NA | 2 |  |  |  |
| ABCB1 | rs1045642 | C | 133 |  | 0.028 | 0.867 |
|  |  | TC | 317 |  |  |  |
|  |  | T | 194 |  |  |  |
|  |  | NA | 15 |  |  |  |
| CYP1B1 | rs1056836 | G | 157 |  | 1.817 | 0.178 |
|  |  | GC | 310 |  |  |  |
|  |  | C | 189 |  |  |  |
|  |  | NA | 3 |  |  |  |
| CYP2D6 | rs1065852 | C | 408 |  | 27.826 | **1.328E-07** |
|  |  | CT | 218 |  |  |  |
|  |  | T | 0 |  |  |  |
| GSTP1 | rs1138272 | C | 537 |  | 4.897 | 0.027 |
|  |  | TC | 110 |  |  |  |
|  |  | T | 12 |  |  |  |
|  |  | NA | 0 |  |  |  |
| ABCG2 | rs2231142 | C | 522 |  | 0.074 | 0.785 |
|  |  | CA | 128 |  |  |  |
|  |  | A | 7 |  |  |  |
|  |  | NA | 2 |  |  |  |
| CYP2B6 | rs2279343 | G | 52 |  | 457.206 | **1.949E-101** |
|  |  | GA | 586 |  |  |  |
|  |  | A | 1 |  |  |  |
| MDM2 | rs2279744 | G | 94 |  | 1.547 | 0.214 |
|  |  | GT | 287 |  |  |  |
|  |  | T | 270 |  |  |  |
|  |  | NA | 8 |  |  |  |
| RWDD3 | rs2296308 | G | 496 |  | 0.177 | 0.674 |
|  |  | GT | 150 |  |  |  |
|  |  | T | 13 |  |  |  |
|  |  | NA | 0 |  |  |  |
| ABCC4 | rs9561778 | G | 415 |  | 0.030 | 0.863 |
|  |  | GT | 215 |  |  |  |
|  |  | T | 29 |  |  |  |
|  |  | NA | 0 |  |  |  |
| SLCO1B3 | rs11045585 | G | 12 |  | 0.255 | 0.614 |
|  |  | GA | 165 |  |  |  |
|  |  | A | 480 |  |  |  |
|  |  | NA | 2 |  |  |  |
| ABCC2 | rs12762549 | C | 163 |  | 7.567 | 0.006 |
|  |  | CG | 364 |  |  |  |
|  |  | G | 132 |  |  |  |
|  |  | NA | 0 |  |  |  |
