# Supplementary material for: Independent replication of polymorphisms predicting toxicity in breast cancer patients randomized between dose-dense and docetaxel-containing adjuvant chemotherapy
Source: Oncotarget. 2017 Nov 27;8(69):113531–42. doi: 10.18632/oncotarget.22697 (PMC5768344; doi:10.18632/oncotarget.22697)
Supplement: Supplementary file 3 [file oncotarget-08-113531-s003.docx]

**Supplementary Table 5:** Summary of the original association studies for anemia **(A)**, febrile neutropenia **(B)** and peripheral neuropathy **(C)**

**A**

| Variable | Groups | Genetic source | Method | Treatment | Endpoint | Reference | Highest risk |
| --- | --- | --- | --- | --- | --- | --- | --- |
| Age at randomization | ≥ 65 years  *vs*  < 65 years |  |  | CEF, CAF, CMF, AC, FAC, FEC, AC-paclitaxel | Hemoglobin concentration < 100 g/L | Dranitsaris et al. Lancet Oncol. 2005 | Age ≥ 65 years |
| Baseline hemoglobin level | Continuous |  |  | CEF, CAF, CMF, AC, FAC, FEC, AC-paclitaxel | Hemoglobin concentration < 100 g/L | Dranitsaris et al. Lancet Oncol. 2005 | Low baseline hemoglobin level (no cut off) |
| Baseline platelet count | ≤ 200x10^9^ cells/L  vs  > 200x10^9^ cells/L |  |  | CEF, CAF, CMF, AC, FAC, FEC, AC-paclitaxel | Hemoglobin concentration < 100 g/L | Dranitsaris et al. Lancet Oncol. 2005 | Baseline platelet count ≤ 200x10(9) cells/L |
| FGFR4 (rs351855) | CC  *vs*  CT/TT | Germline DNA | Sequenom MassARRAY | 3-6 cycles of FEC or FEC+docetaxel | Grade 3-4 anemia (CTC 3.0) | Vulsteke et al. Ann Oncol. 2013 | CC genotype |
| ABCB1 (MDR1; rs1045642) | TT  *vs*  TC/CC | Whole blood DNA | ABI Prism 3730 genetic analyzer | docetaxel-containing chemotherapy | Any grade anemia | Choi et al.  Cancer Res Treat. 2015 | TT genotype |
| ABCC4  (rs9561778) | GG  *vs*  GT/TT | Whole blood DNA | PCR-RFLP | cyclophosphamide-containing chemotherapy | Grade 3-4 anemia | Islam et al. Tumour Biol. 2015 | GT or TT genotypes |

**B**

| Variable | Groups | Genetic source | Method | Treatment | Endpoint | Reference | Highest risk |
| --- | --- | --- | --- | --- | --- | --- | --- |
| Age at randomization | ≥ 65 years  *vs*  < 65 years |  |  | Multiple regimens, containing doxorubicin and/or cyclophosphamide and/or docetaxel | FN: oral temperature ≥ 38.3 °C and neutrophil count of <500 cells/mm^2^ | Aapro et al. Eur J Cancer. 2006 | Age ≥ 65 years |
| Baseline hemoglobin level | Continuous |  |  | 3-6 cycles of FEC or FEC+docetaxel | FN: ANC < 0.5x10^9^/L and body temperature  ≥ 38°C | Pfeil et al. BMC Cancer. 2014 | Low hemoglobin level (no cut off) |
|  | Continuous |  |  | Dose-dense AC-paclitaxel | Grade 3-4 hematologic toxicity | Zauderer et al. Breast Cancer Res Treat. 2009 | Low hemoglobin level (no cut off) |
| Absolut neutrophil count | ≤ 3.1x10^9^ cell/L  *vs*  > 3.1x10^9^ cell/L |  |  | 6 cycles FEC | FN (undefined) | Jenkins et al. Ann Oncol. 2009 | Low neutrophil count (≤ 3.1x10^9^ cell/L) |
| Baseline platelet count | Continuous |  |  | 3-6 cycles of FEC or FEC+docetaxel | FN: ANC < 0.5x10^9^/L and body temperature  ≥ 38°C | Pfeil et al. BMC Cancer. 2014 | Low platelet count (no cut off) |
|  | Continuous |  |  | 6 cycles FEC | FN | Jenkins et al. Ann Oncol. 2009 | Low platelet count was significant risk factor for FN in BC patients |
| Creatinine | Continuous |  |  | cyclophosphamide, methotrexate and fluorouracil (CMF) or anthracycline-based regimen | Grade 3-4 hematologic toxicity | Hurria et al. Drugs Aging. 2005 | Increased creatinine |
| GSTP1 (rs1695) | AG (rs1695) and CC (rs1138272)  *vs*  other genotypes | Whole blood DNA | PCR-RFLP | docetaxel | FN: temperature ≥ 38.5 °C twice and neutrophil count <1.0x10^9^/L | Tran et al. Clin Pharmacol Ther. 2006 | AG genotype of rs1695 and CC genotype of rs1138272 |
|  | AA  *vs*  AG/GG | FFPE tissue of normal lymph node | Sequenom MALDI-TOF mass spectrometry | 6 cycles of CAF or CMF | Grade 3-4 neutropenia (<1,000/mm3 | Yao et al. Clin Cancer Res. 2010 | AA genotype |
|  |  | Whole blood DNA | TaqMan SNP Genotyping Assays | 4 or 6 cycles of FEC100 or 4 cycles of EC | FN: temperature > 38 °C and ANC < 1000/µl | Sugishita et al. Breast Cancer. 2016 | AA genotype |
| FGFR4 (rs351855) | CC/CT  *vs*  TT | Germline DNA | Sequenom MassARRAY | 3-6 cycles of FEC or FEC+docetaxel | FN: ANC < 0.5x10^9^/L and body temperature  ≥ 38°C | Pfeil et al. BMC Cancer. 2014 | TT lower risk of FN than TC or CC in BC patients |
|  | CC/CT  *vs*  TT | Normal and tumor tissue | TaqMan OpenArray technology | 6 cycles of TAC | FN according to NCI-CTCAE version 4.0 | Charehbili et al. Pharmacogenomics. 2015 | TT genotype |
| CYP3A5 (rs7767746) | GG  vs  GA/AA | Whole blood DNA | LightCycler®480 Real-Time PCR system | 4 cycles of AC | Grade 4 neutropenia (neutrophil count <500/µl) | Tang et al. J Cancer Res Clin Oncol. 2013 | AA and GA genotypes |
| ABCB1 (MDR1; rs1045642) | TT  *vs*  TC/CC | Whole blood DNA | ABI Prism 3730 genetic analyzer | docetaxel-containing chemotherapy | Any grade neutropenia | Choi et al. Cancer Res Treat. 2015 | TT genotype |
|  |  | Whole blood DNA | PCR-RFLP | docetaxel | Grade 3 neutropenia | Tran et al. Clin Pharmacol Ther. 2006 | TT genotype |
| CYP1B1 (rs1056836) | CC  *vs*  CG/GG | Whole blood DNA | PCR-RFLP | 4 cycles 5-FU, doxorubicin/epirubicin and cyclophosphamide followed by paclitaxel or docetaxel | Dose reduction/delay due to neutropenia | Tulsyan et al. Gene. 2014 | Haplotype with ABCB1 (rs1045642) |
| ABCG2 (rs2231142) | CC  *vs*  CA/AA | Whole blood DNA | METPlus arrays Affimetrix | AC or FAC followed by 3-6 cycles of docetaxel with or without trastuzumab | FN | Awada et al. OMICS. 2013 | CC genotype |
| MDM2 (rs2279744) | TT/TG  *vs*  GG | Genomic DNA from peripheral monocytes | TaqMan SNP Genotyping Assays | 6 cycles of FEC | Severe neutropenia: neutrophil count <100/mm^3^ | Okishiro et al. Breast Cancer Res Treat. 2012 | TT and TG genotypes |
| ABCC4  (rs9561778) | GG  *vs*  GT/TT | Genomic DNA | multiplex PCR-invader assay or direct sequencing | Cyclophosphamide-containing chemotherapy | Grade 3-4 neutropenia | Low et al. J Hum Genet. 2009 | TT genotype |
| SLCO1B3 (rs11045585) | AA  *vs*  AG/GG | Genomic DNA | multiplex PCR-invader assay or direct sequencing | Docetaxel-containing chemotherapy | Grade 3-4 neutropenia | Kiyotani et al. Cancer Sci. 2008 | AG and GG genotypes |
| ABCC2 (rs12762549) | CC  vs  CG/GG | Genomic DNA | multiplex PCR-invader assay or direct sequencing | docetaxel | Grade 3-4 neutropenia | Kiyotani et al. Cancer Sci. 2008 | CG and GG genotypes |

**C**

| Variable | Groups | Genetic source | Method | Treatment | Endpoint | Reference | Highest risk |
| --- | --- | --- | --- | --- | --- | --- | --- |
| Age at randomization | Continuous |  |  | AC-paclitaxel, AC+bevacizumab followed by paclitaxel+ bevacizumab, AC+ bevacizumab followed by paclitaxel+ bevacizumab followed by bevacizumab | Grade 2-4 neuropathy | Schneider et al. J Clin Oncol. 2011 | Higher age (no cut off) |
| Diabetes | Yes  *vs*  No |  |  | Docetaxel or paclitaxel single agent or in combination with other agents | Dose reductions | Bhatnagar et al. Springerplus. 2014 | Pre-existence of diabetes |
| GSTP1 (rs1695) | AA  vs  AG/GG | Genomic DNA from peripheral lymphocytes | PCR-RFLP | Docetaxel | Grade 2-4 docetaxel-induced peripheral neuropathy | Mir et al. Ann Oncol. 2009 | AA genotype |
| TECTA (rs1829) | CC/CT  vs  TT | Germline DNA | Infinium Human Omni1 array | AC + placebo or bevacizumab followed by paclitaxel and, in some cases, bevacizumab | Grade 2-4 neuropathy | Schneider et al. J Clin Oncol. 2011 | TT genotype |
| GSTP1 (rs1138272) | CC  vs  CT/TT | Whole blood DNA | TaqMan SNP Genotyping Assays | 3 cycles of EC followed by 3 cycles of docetaxel or 6 ccyles of cyclophosphamide/docetaxel | Grade 2-4 docetaxel-induced peripheral neuropathy | Eckhoff et al. Acta Oncol. 2015 | TT and CT genotypes |
| RWDD3 (rs2296308) | GG/GT  vs  TT | Germline DNA | Infinium Human Omni1 array | AC + placebo or bevacizumab followed by paclitaxel and, in some cases, bevacizumab | Grade 2-4 neuropathy | Schneider et al. J Clin Oncol. 2011 | TT genotype |
