# Supplementary material for: Independent replication of polymorphisms predicting toxicity in breast cancer patients randomized between dose-dense and docetaxel-containing adjuvant chemotherapy
Source: Oncotarget. 2017 Nov 27;8(69):113531–42. doi: 10.18632/oncotarget.22697 (PMC5768344; doi:10.18632/oncotarget.22697)
Supplement: Supplementary file 4 [file oncotarget-08-113531-s004.docx]

**Supplementary Table 6:** Validation of previously reported associations between anemia **(A1)**, febrile neutropenia **(B1)** and peripheral neuropathy **(C1)** and SNPs using univariate binary logistic regression analyses. Multivariate binary logistic regression analyses **(A2, C2)** were made with only the significantly different factors. OR = odds ratio; CI = confidence interval

**A1**

|  |  |  | MATADOR study | | |
| --- | --- | --- | --- | --- | --- |
| Factor | **Groups** | **Reference** | **No. of patients with toxicity (%)** | **All patients** | |
|  |  |  |  | **OR** | **95% CI** |
| Age | < 65 years  vs  ≥ 65 years | Dranitsaris | 68/616 (11.0)  9/30 (30.0) | **3.45** | **1.52-7.85** |
| Baseline platelet count | > 200x10^9^ cells/L  vs  ≤ 200x10^9^ cells/L | Dranitsaris | 63/585 (10.8)  14/55 (25.5) | **2.83** | **1.46-5.48** |
| FGFR4 (rs351855) | CC  *vs*  CT/TT | Vulsteke | 32/304 (10.5)  42/334 (12.6) | 1.22 | 0.75-1.99 |
| ABCB1 (rs1045642) | TT/TC  *vs*  CC | Choi | 58/497 (11.7)  17/130 (13.1) | 1.14 | 0.64-2.03 |
| ABCC4  (rs9561778) | GG  *vs*  GT/TT | Islam | 54/403 (13.4)  22/239 (9.2) | 0.66 | 0.39-1.11 |

**A2**

|  |  |  | MATADOR study | | |
| --- | --- | --- | --- | --- | --- |
| Factor | **Groups** | **Reference** | **No. of patients with toxicity (%)** | **All patients** | |
|  |  |  |  | **OR** | **95% CI** |
| Age | < 65 years  vs  ≥ 65 years | Dranitsaris | 68/616 (11.0)  9/30 (30.0) | **2.99** | **1.28-7.02** |
| Baseline platelet count | > 200x10^9^ cells/L  vs  ≤ 200x10^9^ cells/L | Dranitsaris | 63/585 (10.8)  14/55 (25.5) | **2.48** | **1.25-4.89** |

**B1**

|  |  |  | MATADOR study | | |
| --- | --- | --- | --- | --- | --- |
| Factor | **Groups** | **Reference** | **No. of patients with toxicity (%)** | **All patients** | |
|  |  |  |  | **OR** | **95% CI** |
| Baseline ANC ANC | >3.1 x 10^9^ cells/L  vs  ≤ 3.1 x 10^9^ cells/L | Jenkins | 56/514 (10.9)  19/126 (15.1) | 1.45 | 0.83-2.55 |
| GSTP1 (rs1695) | other genotypes  vs  AG (rs1695) and CC (rs1138272) | Tran | 44/429 (10.3)  30/209 (14.4) | 1.47 | 0.89-2.41 |
|  | AA  *vs*  AG/GG | Sugishita  Yao | 27/268 (10.1)  47/370 (12.7) | 1.30 | 0.79-2.15 |
| FGFR4 (rs351855) | CC/CT  *vs*  TT | Pfeil  Charehbili | 66/579 (11.4)  8/59 (13.6) | 1.22 | 0.55-2.68 |
| CYP3A5 (rs776746) | GG  vs  GA/AA | Tang | 57/532 (10.7)  18/108 (16.7) | 1.67 | 0.94-2.96 |
| ABCB1 (rs1045642) | TT  *vs*  TC/CC | Choi  Tran | 23/190 (12.1)  50/437 (11.4) | 0.94 | 0.55-1.60 |
| ABCB1  CYP1B1  (rs1045642*  rs1056836) |  | Tulsyan |  | 0.64 | 0.38-1.06 |
| ABCG2 (rs2231142) | CC  *vs*  CA/AA | Awada | 56/509 (11.0)  19/131 (14.5) | 1.37 | 0.78-2.40 |
| MDM2  (rs2279744) | TT/TG  *vs*  GG | Okishiro | 64/543 (11.8)  8/91 (8.8) | 0.72 | 0.33-1.56 |
| ABCC4  (rs9561778) | GG  *vs*  GT/TT | Low | 54/403 (13.4)  21/239 (8.8) | 0.62 | 0.37-1.06 |
| SLCO1B3 (rs11045585) | AA  *vs*  AG/GG | Kiyotani | 55/465 (11.8)  20/175 (11.4) | 0.96 | 0.56-1.66 |
| ABCC2 (rs12762549) | CC/CG  vs  GG | Kiyotani | 59/515 (11.5)  16/127 (12.6) | 1.11 | 0.62-2.01 |

**C1**

|  |  |  | MATADOR study | | |
| --- | --- | --- | --- | --- | --- |
| Factor | **Groups** | **Reference** | **No. of patients with toxicity (%)** | **All patients** | |
|  |  |  |  | **OR** | **95% CI** |
| diabetes | no  vs  yes | Bhatnagar | 58/643 (9.1)  2/11 (18.2) | 2.21 | 0.47-10.46 |
| GSTP1 (rs1695) | AA  vs  AG/GG | Mir | 20/268 (7.5)  40/370 (10.8) | 1.50 | 0.86-2.64 |
| TECTA (rs1829) | CC/CT  vs  TT | Schneider | 52/608 (8.6)  9/32 (28.1) | **4.18** | **1.84-9.51** |
| GSTP1 (rs1138272) | CC  vs  CT/TT | Eckhoff | 43/525 (8.2)  18/117 (15.4) | **2.04** | **1.13-3.68** |
| RWDD3 (rs2296308) | GG/GT  vs  TT | Schneider | 61/630 (9.7)  0/12 (0.0) | 0.00 | 0.00 |

**C2**

|  |  |  | MATADOR study | | |
| --- | --- | --- | --- | --- | --- |
| Factor | **Groups** | **Reference** | **No. of patients with toxicity (%)** | **All patients** | |
|  |  |  |  | **OR** | **95% CI** |
| TECTA (rs1829) | CC/CT  vs  TT | Schneider | 52/608 (8.6)  9/32 (28.1) | **4.51** | **1.96-10.37** |
| GSTP1 (rs1138272) | CC  vs  CT/TT | Eckhoff | 43/525 (8.2)  18/117 (15.4) | **2.19** | **1.20-3.99** |
