# Supplementary material for: Independent replication of polymorphisms predicting toxicity in breast cancer patients randomized between dose-dense and docetaxel-containing adjuvant chemotherapy
Source: Oncotarget. 2017 Nov 27;8(69):113531–42. doi: 10.18632/oncotarget.22697 (PMC5768344; doi:10.18632/oncotarget.22697)
Supplement: Supplementary file 5 [file oncotarget-08-113531-s005.docx]

**Supplementary Table 7:** Risk of anemia **(A)**, febrile neutropenia **(B)**and peripheral neuropathy **(C)**per treatment arm in previously reported clinical or genotype subgroups. OR = odds ratio; CI = confidence interval

**A**

| Factor | Groups | Reference | ddAC | TAC | TAC vs ddAC | Test for interaction |
| --- | --- | --- | --- | --- | --- | --- |
|  |  |  | **No. of patients with toxicity (%)** | **No. of patients with toxicity (%)** | **OR (95% CI)** | **p-value** |
| Age | < 65 years  vs  ≥ 65 years | Dranitsaris | 56/310 (18.1)  6/17 (35.3) | 12/306 (3.9)  3/13 (23.1) | **0.19 (0.10-0.35)**  0.55 (0.11-2.81) | 0.223 |
| Baseline platelet count | > 200x10^9^ cells/L  vs  ≤ 200x10^9^ cells/L | Dranitsaris | 52/294 (17.7)  10/28 (35.7) | 11/291 (3.8)  4/27 (14.8) | **0.18 (0.09-0.36)**  0.31 (0.08-1.16) | 0.475 |
| FGFR4 (rs351855) | CC  *vs*  CT/TT | Vulsteke | 25/145 (17.2)  34/178 (19.1) | 7/159 (4.4)  8/156 (5.1) | **0.22 (0.09-0.53)**  **0.23 (0.10-0.51)** | 0.954 |
| ABCB1 (rs1045642) | TT/TC  *vs*  CC | Choi | 47/256 (18.4)  13/62 (21.0) | 11/241 (4.6)  4/68 (5.9) | **0.21 (0.11-0.42)**  **0.24 (0.07-0.77)** | 0.883 |
| ABCC4  (rs9561778) | GG  *vs*  GT/TT | Islam | 42/203 (20.7)  19/122 (15.6) | 12/200 (6.0)  3/117 (2.6) | **0.25 (0.13-0.48)**  **0.14 (0.04-0.50)** | 0.456 |

**B**

| Factor | Groups | Reference | ddAC | TAC | TAC vs ddAC | Test for interaction |
| --- | --- | --- | --- | --- | --- | --- |
|  |  |  | **No. of patients with toxicity (%)** | **No. of patients with toxicity (%)** | **OR (95% CI)** | **p-value** |
| Baseline ANC ANC | >3.1 x 10^9^ cells/L  vs  ≤ 3.1 x 10^9^ cells/L | Jenkins | 24/256 (9.4)  11/66 (16.7) | 32/258 (12.4)  8/60 (13.3) | 1.37 (0.78-2.40)  0.77 (0.29-2.06) | 0.319 |
| GSTP1 (rs1695) | other genotypes  vs  AG (rs1695) and CC (rs1138272) | Tran | 23/211 (10.9)  12/112 (10.7) | 21/218 (9.6)  18/97 (18.6) | 0.87 (0.47-1.63)  1.90 (0.86-4.17) | 0.129 |
|  | AA  *vs*  AG/GG | Sugishita  Yao | 15/138 (10.9)  20/185 (10.8) | 12/130 (9.2)  27/185 (14.6) | 0.83 (0.38-1.86)  1.41 (0.76-2.62) | 0.309 |
| FGFR4 (rs351855) | CC/CT  *vs*  TT | Pfeil  Charehbili | 28/293 (9.6)  7/30 (23.3) | 38/286 (13.3)  1/29 (3.4) | 1.45 (0.86-2.43)  0.12 (0.01-1.02) | **0.027** |
| CYP3A5 (rs776746) | GG  vs  GA/AA | Tang | 29/271 (10.7)  6/52 (11.5) | 28/261 (10.7)  12/56 (21.4) | 1.00 (0.58-1.74)  2.09 (0.72-6.06) | 0.229 |
| ABCB1 (rs1045642) | TT  *vs*  TC/CC | Choi  Tran | 10/95 (10.5)  23/223 (10.3) | 13/95 (13.7)  27/214 (12.6) | 1.35 (0.56-3.24)  1.26 (0.70-2.27) | 0.896 |
| ABCG2 (rs2231142) | CC  *vs*  CA/AA | Awada | 28/264 (10.6)  7/59 (11.9) | 28/245 (11.4)  12/72 (16.7) | 1.09 (0.62-1.90)  1.49 (0.55-4.05) | 0.594 |
| MDM2  (rs2279744) | TT/TG  *vs*  GG | Okishiro | 33/277 (11.9)  2/46 (4.3) | 31/266 (11.7)  6/45 (13.3) | 0.98 (0.58-1.64)  3.39 (0.65-17.75) | 0.160 |
| ABCC4  (rs9561778) | GG  *vs*  GT/TT | Low | 24/203 (11.8)  11/122 (9.0) | 30/200 (15.0)  10/117 (8.5) | 1.32 (0.74-2.34)  0.94 (0.39-2.31) | 0.540 |
| SLCO1B3 (rs11045585) | AA  *vs*  AG/GG | Kiyotani | 25/240 (10.4)  10/85 (11.8) | 30/225 (13.3)  10/90 (11.1) | 1.32 (0.75-2.33)  0.94 (0.37-2.38) | 0.535 |
| ABCC2 (rs12762549) | CC/CG  vs  GG | Kiyotani | 26/267 (9.7)  9/58 (15.5) | 33/248 (13.3)  7/69 (10.1) | 1.42 (0.82-2.46)  0.62 (0.21-1.77) | 0.167 |

**C**

| Factor | Groups | Reference | ddAC | TAC | TAC vs ddAC | Test for interaction |
| --- | --- | --- | --- | --- | --- | --- |
|  |  |  | **No. of patients with toxicity (%)** | **No. of patients with toxicity (%)** | **OR (95% CI)** | **p-value** |
| diabetes | no  vs  yes | Bhatnagar | 15/323 (4.6)  0/4 (0) | 43/311 (13.8)  2/7 (28.6) | **3.30 (1.79-6.06)**  - | 0.999 |
| GSTP1 (rs1695) | AA  vs  AG/GG | Mir | 4/138 (2.9)  11/185 (5.9) | 16/130 (12.3)  29/185 (15.7) | **4.70 (1.53-14.46)**  **2.94 (1.42-6.08)** | 0.492 |
| TECTA (rs1829) | CC/CT  vs  TT | Schneider | 14/315 (4.4)  1/9 (11.1) | 38/293 (13.0)  8/23 (34.8) | **3.20 (1.70-6.05)**  4.27 (0.45-40.44) | 0.810 |
| GSTP1 (rs1138272) | CC  vs  CT/TT | Eckhoff | 11/275 (4.0)  4/50 (8.0) | 32/250 (12.8)  14/67 (20.9) | **3.52 (1.74-7.15)**  3.04 (0.93-9.88) | 0.833 |
| RWDD3 (rs2296308) | GG/GT  vs  TT | Schneider | 15/317 (4.7)  0/8 (0) | 46/313 (14.7)  0/4 (0) | **3.47 (1.89-6.36)**  - | 1.000 |
